# Analysis of protein-protein interaction network based on transcriptome profiling of ovine granulosa cells identifies candidate genes in cyclic recruitment of ovarian follicles

**DOI:** 10.1186/s40781-018-0171-y

**Published:** 2018-06-11

**Authors:** Reza Talebi, Ahmad Ahmadi, Fazlollah Afraz

**Affiliations:** 1 Department of Animal Sciences, Faculty of Agriculture, Bu-Ali Sina University, Hamedan, Iran; 2 Department of Livestock and Aquaculture Biotechnology, Agricultural Biotechnology Research Institute of North Region, Rasht, Iran

**Keywords:** Protein–protein interaction network, Biomarkers, Ovarian follicles, Granulosa cells, Cyclic recruitment

## Abstract

After pubertal, cohort of small antral follicles enters to gonadotrophin-sensitive development, called recruited follicles. This study was aimed to identify candidate genes in follicular cyclic recruitment via analysis of protein-protein interaction (PPI) network. Differentially expressed genes (DEGs) in ovine granulosa cells of small antral follicles between follicular and luteal phases were accumulated among gene/protein symbols of the Ensembl annotation. Following directed graphs, *PTPN6* and *FYN* have the highest indegree and outdegree, respectively. Since, these hubs being up-regulated in ovine granulosa cells of small antral follicles during the follicular phase, it represents an accumulation of blood immune cells in follicular phase in comparison with luteal phase. By contrast, the up-regulated hubs in the luteal phase including *CDK1*, *INSRR* and *TOP2A* which stimulated DNA replication and proliferation of granulosa cells, they known as candidate genes of the cyclic recruitment.

**Electronic supplementary material:**

The online version of this article (10.1186/s40781-018-0171-y) contains supplementary material, which is available to authorized users.

## Introduction

Small antral follicles represent the fundamental developmental unit of the mammalian ovary and, as such, serve the needs of the entire reproductive life span. After pubertal, cohort of small antral follicles enters to gonadotrophin-sensitive development upon FSH (Follicle Stimulating Hormone) stimulation, called cyclic recruited follicles [1, 2]. Cyclic recruitment, although obligatory, does not guarantee ovulation because growing follicles are vulnerable to atresia and thus may fall out from the growth trajectory [3]. Among somatic cells of the antral follicles, granulosa cells will undergo serious changes morphologically and physiologically during the processes of proliferation, differentiation, ovulation, lutenization and atresia [4].

To understand gene function of the cellular processes, genes must be studied in the context of networks. Emerging tools from systems biology going beyond simple gene ontology (GO) terms like causal network modelling linking gene expression analysis to gene interaction information are sorely needed [5, 6]. Nowadays, biological networks allow a comprehensive examination of the complex mechanisms for targeted empirical studies [7]. Numerous studies have been conducted in attempt to identify candidate genes of reproductive biology via gene networks analysis such as development of rat primordial follicles [8, 9], early embryo development in mouse [10] and bovine [11], gene networks of bovine oocytes [12] and bovine granulosa cells of small and large follicles [13]. Protein-protein interaction (PPI) networks are one the most known approaches for representation of candidate genes/proteins beyond of high-throughput studies [13–19].

In previous study, we surprisingly have shown differences in transcriptome profiles of ovine (sheep) granulosa cells between small antral follicles (1–3 mm) collected during the follicular and luteal phases [20]. Therefore, the specific purpose of the present study was to survey the differentially expressed genes (DEGs) from the previous study using the analysis of PPI networks in order to identification of candidate genes that probably those are impressive in cyclic recruitment of ovarian follicles.

## Materials and methods

### Dataset assessment

The main dataset of the present study was belonged to 663 DEGs in ovine granulosa cells between small antral follicles (1–3 mm in diameter) collected during the follicular and luteal phases [20]. These DEGs afterwards were annotated using a standard Ensembl gene annotation system.

### Construction of protein-protein interaction (PPI) network

A large PPI network was reconstructed from the 646 genes/protein symbols (Additional file 1) of the Ensembl annotation (Ovis_aries.Oar_v3.1.91). In order to map pairwise interactions, all computational methods in STRING database Version. 10.0 [21] containing neighborhoods, gene fusion, co-occurrence, homology, co-expression, experiments, databases and text mining, were utilized with the medium confidence score (> 0.4).

### Network analysis and modules selection

Cytoscape Version 3.1.1 [22] was used to plot and analyze the centralities, clustering and modularity of the PPI network. The MCODE (Molecular Complex Detection) v1.4.0-beta2 was performed to screen modules of PPI network with degree cutoff = 2, node score cutoff = 0.2, k-core = 2, and max. Depth = 100 [14]. This is a well-known automated algorithm to find highly interconnected subgraphs that detects densely connected regions in large PPI networks that may represent molecular complexes [23]. Also, the functional modules were chosen with the number of nodes ≥16 and nodes score [19].

### Enrichment analysis for the functional modules

Biological significance of these predicted modules were inferred by ClueGO [24] plugin of Cytoscape. The statistical significance of the biological terms analyzed was calculated with Right-sided enrichment hypergeometric test and Benjamini and Hochberg *P*-value correction [25] to reduce false positives- and negatives. Kappa statistics were used to link and grouping of the enriched terms and functional grouping of them as described by Bindea et al. [24]. The minimum connectivity of the pathway network (kappa score) was set to 0.4 units.

### Identification of hub genes/proteins and its directed interaction network

In this study, all networks were utilized to identify hub genes/proteins which important in folliculogenesis during the ovine estrous cycle. Hubs were detected by calculating the node degree distribution [16] using the Network Analyzer (http://apps.cytoscape.org/apps/networkanalyzer) plugin of Cytoscape [24]. Degree gives a simple count of the number of interactions of a given node [26]. Additionally, we utilized several centrality parameters including stress, betweenness and closeness instead of using degree centrality itself. The mathematical formulas for the calculation of centrality parameters including stress, betweenes and clossness, are available in Additional file 2 that has been retrieved from Zhuang et al. [26]. By extract direction of PPI from STRING Version. 10.0 [21], a directed gene network was reconstructed from the hub genes/proteins. These important proteins were extracted from hub genes/proteins via network analysis and modularity of the network. This small, but important, graph is visualized by Cytoscape Version 3.1.1 [22]. Using CluePedia v.1.1.7. [24] plugin of Cytoscape, hub genes/proteins were placed in a directed gene network based on its molecular function.

## Result and discussion

**Fig. 1:**
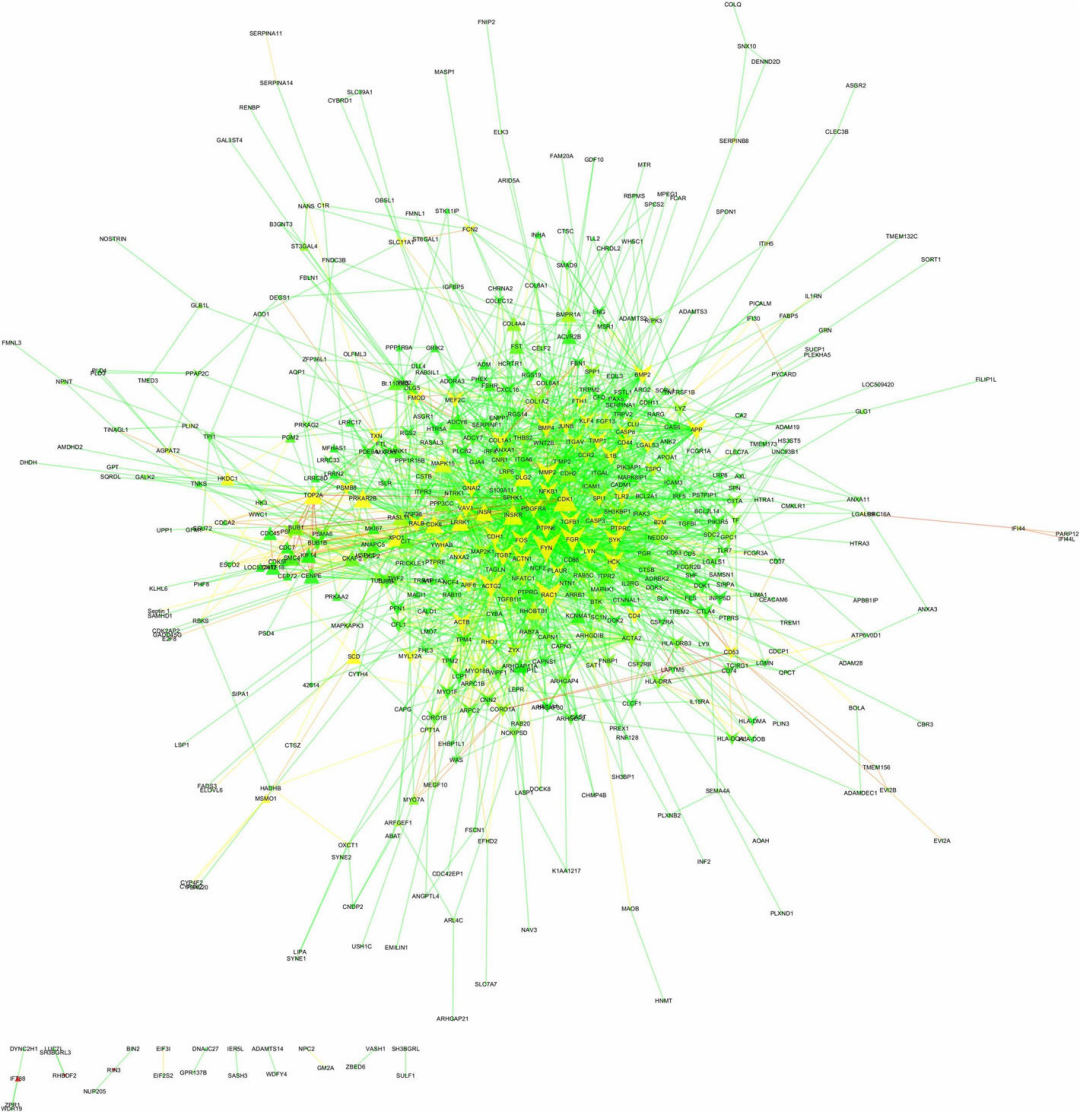
The Un-directed PPI network for total genes/proteins differentially expressed (cut-off of *P* < 0.05 in corrected tests of Benjamini-Hochberg) in luteal vs. follicular phases of ovine estrous cycle. The PPI network illustrates degree (node size) and betweenness centrality (node color) and layout option of edge weighted spring embedded. Up- and down-regulated genes in luteal phase vs. follicular phase are shown with triangles and V shapes, respectively

Among 646 DEGs (Additional file 1), 498 proteins were annotated on the Total PPI network. Also, 2191 edges containing neighborhoods, gene fusion, co-occurrence, homology, co-expression, experiments, databases and text mining, were interacted between such genes/proteins (Fig. 1 and Additional file 3). The statistics of network containing network density, network diameter and clustering coefficient were 0.018, 9 and 0.275, respectively. The power law of degree distribution was followed with an *R*^*2*^ = 0.895. Meanwhile, *R*^*2*^ is computed on logarithmized values. Some proteins in this network had high values in all degree, stress and betweenness centralities, such as *FYN*, *CDK1*, *RAC1*, *ACTG2*, *FGR*, *APP*, *MMP2*, *SYK*, *CDH1*, *TGFB1*, *PTPN6*, *ITGB7*, *FOS*, *ACTN1*, *GNAI2*, *INSRR*, *BMP4*, *BMP2*, *LYN* and *HCK* (Additional file 3). This list was utilized to identify some candidate genes among hubs as major regulators in cyclic recruitment of ovine small antral follicles.

From the total PPI network, thirteen modules were extracted, among which five subnetworks (modules of 1, 2, 4, 6 and 12) were detected with the intra-connection nodes ≥16 and node score > 2.0 (Table 1). A module/subnetwork is a group of closely related proteins that act in concert to perform specific biological functions through PPI network that occurs in time and space [15]. In the biological processes (BP), the highest significant terms for module 1, module 2, module 4, module 6 and module 12 were related to the regulation of B cell receptor signaling pathway, Fc receptor mediated stimulatory signaling pathway, positive regulation of reactive oxygen species metabolic process, innate immune response activating cell surface receptor signaling pathway and positive regulation of DNA replication, respectively (Table 2). As opposite performances among these modules, we have surprisingly identified four modules of 1, 2, 4 and 6, in relevant to immune system in comparison with module 12 in relevant to cell proliferation (Table 2).

**Table 1:** Statistics for thirteen subnetworks identified by MCODE method in PPI network from complete DEGs (cutoff *P* < 0.05 in corrected tests of Benjamini-Hochberg)

Module	Score	Nodes	Interactions	Gene/Protein ID
1	8.941	18	76	GNAI2, BTK, RGS14, LYN, ADORA3, PDGFRA, CXCL16, NEDD9, ADCY6, RGS19, HTR5A, ADCY7, CCR2, APP, ANXA1, CNR1, PTPRC, PTPN6
2	6.087	24	70	CD86, FNBP1, TGFB1I1, VAV1, DOK2, RAC1, CAPN3, HCK, KCNMA1, CENPE, ARRB1, BUB1B, CAPN1, ITPR2, PIK3R5, ITPR3, SYK, BMP2, DLG2, ARHGDIB, CDK6, TOP2A, SH3KBP1, FYN
3	5.6	6	14	CD74, HLA-DRA, HLA-DRB3, HLA-DMA, HLA-DQA1, HLA-DOB
4	4.231	27	55	SERPINF1, NCF2, CYBA, CORO1A, LCP1, PPP3CC, TGFB1, CFL1, PFN1, CDH2, MMP2, INSR, COL6A1, TSPO, TPM2, COL8A1, MAP2K1, CORO1B, DOK1, ICAM1, NFATC1, COL1A1, FOS, KLF4, TAGLN, MYO1F, INPP5D
5	4	4	6	CFD, CLU, GAS6, SERPINA1
6	3.879	34	64	NTN1, CDK5RAP2, ARHGAP4, CKAP5, ICAM3, CDH1, NFKB1, IL1B, ZYX, ANAPC5, ITGAV, COL4A4, MYL12A, SPI1, ITGB7, FBN1, CNN2, MYO18B, FGR, PGR, TLR2, MKI67, ARHGAP30, PSMA6, PSMB8, RHOBTB1, COL1A2, THBS2, RHOJ, KIF14, HMHA1, TPM4, PSMD8, CEP72
7	3.6	6	9	TUBB6, LRRK1, ACTB, RAB7A, RAB10, WIPF1
8	3.333	4	5	PPAP2C, PLD4, PLD3, AGPAT2
9	3	3	3	SERPINB8, SNX10, DENND2D
10	3	3	3	PRKAA2, PRKAR2B, PRKAG2
11	3	3	3	NPNT, TINAGL1, PLIN2
12	2.8	16	21	ACTA2, SMAD9, ARPC2, ENG, CDK1, TIMP1, MAPK15, BUB1, ACVR2B, XPO1, NCKIPSD, ACTN1, INSRR, WAS, CDC45, BMP4
13	2.667	4	4	BCL2L14, BCL2A1, CASP3, PAX8

*Abbreviations*: *PPI* Protein–protein interaction, *DEGs* Differentially expressed genes

**Table 2:** Highly significant terms (*P* < 0.05 in corrected test of Benjamini-Hochberg) in BP enrichment of modules from total PPI network

Category	GO ID	GO Term	Associated Genes/proteins Found	Adjusted *P*-value
Module 1				
BP	GO:0050855	regulation of B cell receptor signaling pathway	LYN, PTPN6, PTPRC	3.44E-06
BP	GO:0050866	negative regulation of cell activation	CCR2, CNR1, LYN, PDGFRA, PTPN6	4.23E-06
BP	GO:0002703	regulation of leukocyte mediated immunity	BTK, CCR2, LYN, PTPN6, PTPRC	4.36E-06
BP	GO:0050853	B cell receptor signaling pathway	BTK, LYN, PTPN6, PTPRC	7.04E-06
BP	GO:0045577	regulation of B cell differentiation	BTK, PTPN6, PTPRC	1.56E-05
Module 2				
BP	GO:0002431	Fc receptor mediated stimulatory signaling pathway	FYN, HCK, ITPR2, ITPR3, RAC1, SYK, VAV1	6.58E-08
BP	GO:0038094	Fc-gamma receptor signaling pathway	FYN, HCK, ITPR2, ITPR3, RAC1, SYK, VAV1	8.67E-08
BP	GO:0002433	immune response-regulating cell surface receptor signaling pathway involved in phagocytosis	FYN, HCK, ITPR2, ITPR3, RAC1, SYK, VAV1	1.68E-07
BP	GO:0038096	Fc-gamma receptor signaling pathway involved in phagocytosis	FYN, HCK, ITPR2, ITPR3, RAC1, SYK, VAV1	1.68E-07
BP	GO:0048010	vascular endothelial growth factor receptor signaling pathway	FYN, ITPR2, ITPR3, RAC1, VAV1	4.86E-06
Module 4				
BP	GO:2000379	positive regulation of reactive oxygen species metabolic process	CYBA, ICAM1, INSR, KLF4, TGFB1, TSPO	1.59E-07
BP	GO:0043200	response to amino acid	CFL1, COL1A1, COL6A1, CYBA, ICAM1, MMP2	2.18E-07
BP	GO:1903426	regulation of reactive oxygen species biosynthetic process	CYBA, ICAM1, INSR, KLF4, TSPO	8.46E-07
BP	GO:2000377	regulation of reactive oxygen species metabolic process	CYBA, ICAM1, INSR, KLF4, TGFB1, TSPO	8.80E-07
BP	GO:0022617	extracellular matrix disassembly	COL1A1, COL6A1, COL8A1, LCP1, MMP2, TGFB1	8.92E-07
Module 6				
BP	GO:0002220	innate immune response activating cell surface receptor signaling pathway	ICAM3, IL1B, NFKB1, PSMA6, PSMB8, PSMD8, TLR2	1.52E-07
BP	GO:0002223	stimulatory C-type lectin receptor signaling pathway	ICAM3, IL1B, NFKB1, PSMA6, PSMB8, PSMD8	2.33E-06
BP	GO:0050764	regulation of phagocytosis	CNN2, FGR, IL1B, ITGAV	8.97E-05
BP	GO:0051437	positive regulation of ubiquitin-protein ligase activity involved in regulation of mitotic cell cycle transition	ANAPC5, PSMA6, PSMB8, PSMD8	1.01E-04
BP	GO:0002479	antigen processing and presentation of exogenous peptide antigen via MHC class I, TAP-dependent	ITGAV, PSMA6, PSMB8, PSMD8	1.06E-04
Module 12				
BP	GO:0045740	positive regulation of DNA replication	BMP4, CDK1, TIMP1	6.24E-05
BP	GO:0090100	positive regulation of transmembrane receptor protein serine/threonine kinase signaling pathway	ACVR2B, BMP4, ENG	9.44E-05
BP	GO:0045446	endothelial cell differentiation	ACVR2B, BMP4, ENG	9.74E-05
BP	GO:0060840	artery development	ACVR2B, BMP4, ENG	1.09E-04

*Abbreviations***:** *BP* Biological process, *PPI* Protein–protein interaction

Among identified hub genes/proteins in Table 3, twenty-five hub genes (*FYN*, *RAC1*, *FGR*, *MMP2*, *SYK*, *LYN*, *TGFB1*, *PTPN6*, *FOS*, *CDH1*, *ITGB7*, *HCK*, *APP*, *ACTN1*, *GNAI2*, *BMP4*, *BMP2*, *PTPRC*, *NFKB1*, *VAV1*, *IL1B*, *COL1A1*, *TIMP1*, *PDGFRA*, *BTK*), were up-regulated in ovine granulosa cells of small antral follicles during the follicular phase (up-regulated genes in Follicular has been shown in Additional file 1). Interestingly, these hubs were significantly connected to immune system and phagocytosis (Table 2). On the contrary, three (*CDK1*, *INSRR* and *TOP2A*) were up-regulated in ovine granulosa cells of small antral follicles during the luteal phase (up-regulated genes in Luteal has been shown in Additional file 1).

**Table 3:** The 28 hub genes/proteins of PPI network

Gene	Degree	Module number	Gene	Degree	Module number	Gene	Degree	Module number	Gene	Degree	Module number
*FYN*	87	2	*TGFB1*	43	2	*ACTN1*	37	12	*VAV1*	34	2
*RAC1*	79	2	*PTPN6*	43	1	*INSRR*	36	12	*IL1B*	29	6
*FGR*	67	6	*FOS*	43	4	*GNAI2*	35	1	*COL1A1*	29	4
*CDK1*	58	12	*CDH1*	42	6	*BMP4*	35	12	*TOP2A*	28	2
*MMP2*	52	4	*ITGB7*	42	6	*BMP2*	35	2	*TIMP1*	28	12
*SYK*	45	2	*HCK*	41	2	*PTPRC*	34	1	*PDGFRA*	28	1
*LYN*	44	1	*APP*	39	1	*NFKB1*	34	6	*BTK*	28	1

*Abbreviations***:** *PPI* Protein–protein interaction

**Fig. 2:**
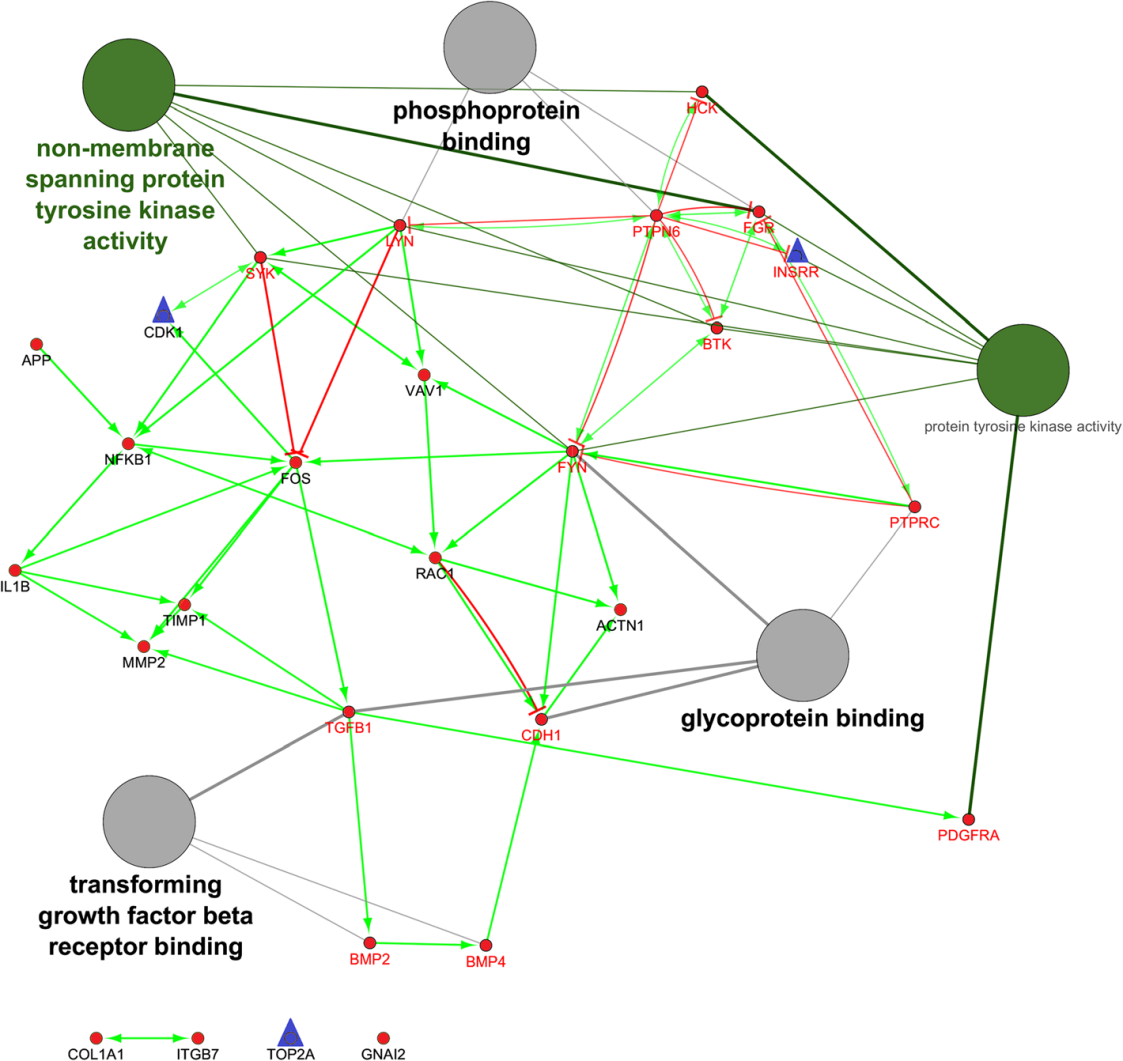
The directed PPI network for the identified hub genes/proteins. The nodes with small red circles and blue triangles are down- and up-regulated hub genes/proteins in luteal phase vs. follicular phase, respectively. The green- and red vectors are revealed the molecular function of activation and inhibition, respectively. The hub genes/proteins in direction with molecular function are shown with red labels

As shown in Fig. 2, the hub genes of *PTPN6* and *FYN* are revealed the highest in-degree and out-degree, respectively (Additional file 4, and Fig. 2). Regarding to protein differential expression in normal tissues from HIPED (the Human Integrated Protein Expression Database), *PTPN6* is overexpressed in Peripheral blood mononuclear cells, Lymph node, Blymphocyte, and Monocytes (http://www.genecards.org/). Moreover, *FYN* is another hub with highest out-degree, is also overexpressed in Peripheral blood mononuclear cells (http://www.genecards.org/). Regardless, *PTPN6* and *FYN* being up-regulated in the ovine granulosa cells of small antral follicles during the follicular phase, represents an accumulation of blood immune cells into small antral follicles of the follicular phase in comparison with luteal phase. Therefore, the protein of FYN can be known as an upstream regulator in inhibition of ovarian folliculogenesis. This protein is belonged to the protein tyrosine kinase (PTK) family as illustrated in regulatory model of molecular function in Fig. 2. These signaling molecules regulate a variety of cellular processes including cell growth, differentiation, mitotic cycle, and oncogenic transformation (http://www.genecards.org/).

As shown in Table 2, the subnetwork 2 with the eight hubs were became the highest in relative to the others. The most significant term of BP in module 2 was belonged to Fc receptor mediated stimulatory signaling for the hub genes/proteins, including *FYN*, *HCK*, *RAC1*, *SYK* and *VAV1* (Table 2). This can regulate immune responses through interacting with Fc receptors [27]. Moreover, the up-regulated hubs of *PDGFRA*, *COL1A1*, *BMP2*, *CDH1*, *ACTN1*, *FOS*, *FYN*, and *TIMP1* in the follicular phase, they also were up-regulated in bovine and porcine granulosa cells of small atretic follicles [28, 29]. By contrast, the up-regulated hubs of *CDK1* and *TOP2A* in the luteal phase, they also were up-regulated in the bovine granulosa cells of small heathy follicles [28]. These genes were cohesively interconnected around up-regulated nodes in classical mitosis checkpoint controllers [30]. Furthermore, *TOP2A* is expressed mainly during the S to G2 /M phase of the cell cycle and is likely to play a major role in DNA catenation during mitosis [31]. Interestingly, such hubs all were belonged to module 12 whose been enriched in positive regulation of DNA, cell cycle and progesterone-mediated oocyte (Table 2). Therefore, the hubs including *CDK1*, *INSRR* and *TOP2A*, represented the proliferation of ovine granulosa cells from small antral follicles during the luteal phase [20], whose probably are crucial in cyclic recruitment of small antral follicles. This is closer to the second theory in recruitment of antral follicles during menstrual cycle by Baerwald et al. [32].

## Conclusion

In this study, we evidenced that cyclic recruitment of small antral follicles mostly occurs in the luteal phase in comparison with follicular phase during the ovine estrous cycle. Based on analysis of PPI network and its modulation, we identified some biomarkers whose potentially impress on cyclic recruitment of ovarian follicles. Surprisingly, *FYN* was identified as upstream regulator that probably inhibits the proliferation of granulosa cells. By contrast, hub genes of *CDK1*, *INSRR* and *TOP2A*, were known as inducers in proliferation of granulosa cells among genes were up-regulated in luteal phase in comparison with follicular phase. These results may provide valuable genetic markers for increasing ewe prolificacy with focus on cyclic recruitment of ovarian small follicles. Nevertheless, further studies using an experimental approach and a greater number of individuals are warranted for the verification of such candidate genes.

## Additional files

Additional file 1: The 646 genes of differentially expressed (DEGs) in luteal vs. follicular phase (*P* < 0.05 in corrected test of Benjamini-Hochberg) from the genes/protein symbols (Additional file 1) of the Ensembl annotation (Ovis_aries.Oar_v3.1.91). The 455 up-regulated genes in ovine granulosa cells of the small antral follicles in the follicular phase. The 191 up-regulated genes in ovine granulosa cells of the small antral follicles in the Luteal phase (Talebi et al. [20]). (XLS 108 kb)

Additional file 2: The mathematical formulas for the analysis of topological parameters like network centrality options such as stress, betweenness and closeness centralities (Zhuang et al. [26]). (DOCX 16 kb)

Additional file 3: The list of 498 proteins as nodes was annotated on the Total PPI network. Edges containing neighborhoods, gene fusion, co-occurrence, homology, coexpression, experiments, databases and text mining, were interacted between such genes/proteins (XLS 839 kb)

Additional file 4: The list of hub genes/proteins from the directed PPI network. The criteria of Indegree and Outdegree have been used for the identification of downstream and upstream regulators in directed PPI network, respectively. (XLS 31 kb)

## Abbreviations

BP: Biological process
CDK1: Cyclin-dependent kinase 1
DEGs: Differentially expressed genes
FYN: Proto-oncogene tyrosine-protein kinase
GO: Gene Oncology
INSRR: Insulin Receptor Related Receptor
PPI: Protein-Protein Interaction
PTPN6: Protein tyrosine phosphatase, non-receptor type 6
TOP2A: Topoisomerase II Alpha
