# Supplementary material for: Analysis of protein-protein interaction network based on transcriptome profiling of ovine granulosa cells identifies candidate genes in cyclic recruitment of ovarian follicles
Source: J Anim Sci Technol. 2018 Jun 11;60:11. doi: 10.1186/s40781-018-0171-y (PMC5994657; doi:10.1186/s40781-018-0171-y)
Supplement: Supplementary file 2 — The mathematical formulas for the analysis of topological parameters like network centrality options such as stress, betweenness and closeness centralities (Zhuang et al. [26]). (DOCX 16 kb) [file 40781_2018_171_MOESM2_ESM.docx]

The other topological parameters like network centrality options such as stress, betweenness and closeness centralities were taken as subsequent indexes for collection of hubs (retrieved from Zhuang et al., 2015).

Zhuang DY, Jiang L, He QQ, Zhou P, Yue T. Identification of hub subnetwork based on topological features of genes in breast cancer. Int J Mol Med. 2015; 35: 664-74.

Stress centrality is considered the number of nodes in the shortest path between two other nodes; the stress is a node centrality index. A ‘stressed’ node is a node traversed by a high number of shortest paths. It is calculated by measuring the number of shortest paths passing through a node. The ‘stress’ [*C_str_* (*v*)] of a node *v* is calculated as follows:

$$Cstr (v)= \sum\sum\sigma st (v)$$

where *σst* (*v*) is the total number of shortest paths from node *s* to node t that pass through *v*.

Betweenness centrality is another topological metric in graphs for determining how the neighbors of a node are interconnected. It reflects the importance of the node based on the number of shortest paths that pass through each node. The betweenness centrality of a node *v* is also calculated as follows:

$$CB(V)= \sum_{s\neq v\neq t\in N} \frac{\delta st(v)}{\delta st}$$

Betweenness centrality of a node scales with the number of pairs of nodes as implied by the summation indices. Therefore, the calculation may be rescaled by dividing the number of pairs of nodes not including *v*, so that *CB*(*v*) ∈ [0,1]. *σst* is the total number of shortest paths from node s to node t.

Closeness centrality is a measure of the average length of the shortest paths to access all other proteins in the network. The larger the value, the more central is the protein. The closeness centrality, *Cc* (*v*) was calculated for each functional category, taking into consideration all the shortest paths for each node. *Cc*(*v*) of node n is defined as the reciprocal of the average shortest path length and is computed as follows (retrieved from 37):

$Cc(v) =\frac{1}{\sum_{t\in N} dG(v,t)}$

where *dG* (*s*, *t*) represents the length of the shortest path between two nodes *s* and t in graph G, which is the sum of the weights of all edges on this shortest path. *dG* (*s*, *s*) = 0, *dG* (*s*, *t*) = *dG* (*t*, *s*) in the undirected graph.
